# Wind tunnel data of the analysis of heat pipe and wind catcher technology for the built environment

**DOI:** 10.1016/j.dib.2015.09.027

**Published:** 2015-10-03

**Authors:** John Kaiser Calautit, Hassam Nasarullah Chaudhry, Ben Richard Hughes

**Affiliations:** a Department of Mechanical Engineering, University of Sheffield, Sheffield S10 2TN, UK; b School of Energy, Geoscience, Infrastructure and Society, Heriot-Watt University, Dubai, United Arab Emirates

## Abstract

The data presented in this article were the basis for the study reported in the research articles entitled ‘Climate responsive behaviour heat pipe technology for enhanced passive airside cooling’ by Chaudhry and Hughes [10] which presents the passive airside cooling capability of heat pipes in response to gradually varying external temperatures and related to the research article “CFD and wind tunnel study of the performance of a uni-directional wind catcher with heat transfer devices” by Calautit and Hughes [1] which compares the ventilation performance of a standard roof mounted wind catcher and wind catcher incorporating the heat pipe technology. Here, we detail the wind tunnel test set-up and inflow conditions and the methodologies for the transient heat pipe experiment and analysis of the integration of heat pipes within the control domain of a wind catcher design.

**Specifications table**

**Table:** 

Subject area	*Environmental Science*
More specific subject area	*Building Engineering, Thermal, Natural Ventilation, Airflow*
Type of data	*Tables, graphs, figure*
How data was acquired	*Hot-wire anemometer (Testo 425), Thermocouple (Type-K, PICO) with data logger (PICO)*
Data format	*Raw data and analysed*
Experimental factors	*Controlled wind tunnel velocity and temperature*
Experimental features	*For the heat pipe analysis, the test was performed at 1:1 scale. For the analysis of the wind catcher, the measurement of the supply and indoor airflow velocity were carried out in a 1:10 scale model of a small room.*
Data source location	*Leeds, United Kingdom*
Data accessibility	*Data is with this article*

**Value of the data**

• The transient heat pipe experimental data can be used by heat pipe CFD model developers when validating their numerical models.
• The wind tunnel data of the wind catcher testing can be used for validating numerical model predictions of the airflow supply rate and air flow distributions in buildings incorporating wind catchers.
• The data can be used to test different turbulence models, boundary conditions, mesh design, discretisation scheme, steady-state and transient simulations etc.
• The value of the data is its use in improving the comparability between the results of other researcher’s models by providing a common benchmark.
• The data can be used to explore different optimisation of the heat pipe arrangement and wind catcher design.

## 1 Data

The data presented in this article is based on the experimental wind tunnel testing of a passive cooling wind catcher detailed in [1], [2], [4]. Heat pipes are incorporated into the design of the wind catcher to improve its thermal performance by reducing the temperature of the supply airflow. The design and performance analysis of the wind catcher are detailed in [1], [2], [3], [4], [5]. The experimental testing is two-fold: first, the heat pipe arrangement is tested inside a wind tunnel test section to establish a transient relationship between the source temperatures and its effect on the passive cooling effectiveness of heat pipes. Second, the heat pipe arrangement is incorporated into a wind catcher and tested inside the same test section to investigate its natural ventilation performance.

The data shared in this article are presented in the supplementary file as follows:

**Wind tunnel inlet flow** (Supplementary Table 1) **–** Measurements of the airflow velocity at the inlet of the wind tunnel are presented in this data file. The measurements were carried out in the inlet region as near as possible to the opening of the test section. This data can be used as inlet velocity boundary condition for the numerical modelling of heat pipes in a control volume and outdoor domain for wind catcher models.

**Heat pipe test** (Supplementary Table 2) **–** The data presented in this file is the transient air temperature formations downstream of the heat pipes in response to the source temperature in both conduction and convection modes. The measurements were recorded at one-second intervals for a total duration of 86,400 s on 24 h.

**Standard wind catcher test** (Supplementary Table 3) **–** This data file presents the indoor and supply airflow velocity measurements for a standard single-sided wind catcher device based on mean airflow velocity measured with hot-wire anemometer. The measurements were carried out below the supply opening of the wind catcher and also inside a test room which was ventilated by the wind catcher.

**Wind catcher with heat pipe test** (Supplementary Table 4) **–** This data file presents the indoor and supply airflow velocity measurements for a uni-directional wind catcher device incorporating the heat pipe arrangement. Similar experimental setup and measurement procedure were employed as the standard wind catcher.

## 2 Experimental methods

### 2.1 Experimental design

Both experimental tests were carried out in a low speed wind tunnel detailed in [5]. The wind tunnel has a test section of the height, width, and length of 0.5 m, 0.5 m, and 1 m. The variable intensity axial fan and 15 kW heating elements were capable of supplying wind speeds up to 13 m/s and air temperatures of up to 60 °C. Full details of the design, specification, operation and characterisation of the wind tunnel is available in [6].

The 1:1 scaled heat pipe experimental investigation was based on establishing a dynamic thermal model to replicate the hourly temperatures for June 21st, 2012 found in hot climatic conditions (i.e. Doha, Qatar). The test-section of the wind tunnel was used as the testing rig for carrying out the experimentation while the cold sink was used as the control volume for the condenser section at the top [6], [7].

Inlet temperatures from the heating elements were varied every 1800 s as per the available climatic data and the thermal performance of the heat pipes were monitored by connecting thermocouples upstream and downstream of the physical domain. The temperatures ranged between 32 °C and 43 °C providing a differential (Δ*T*) of 11 °C for the duration of 24 h or 86,400 s. The temperature of the control volume for the cold sink was maintained between 15 °C and 18 °C to ensure a continuous cyclic operation of the heat pipe cycle as typical operation of heat pipes require a temperature drop of at least 5 °C for correct functionality

**Fig. 1:**
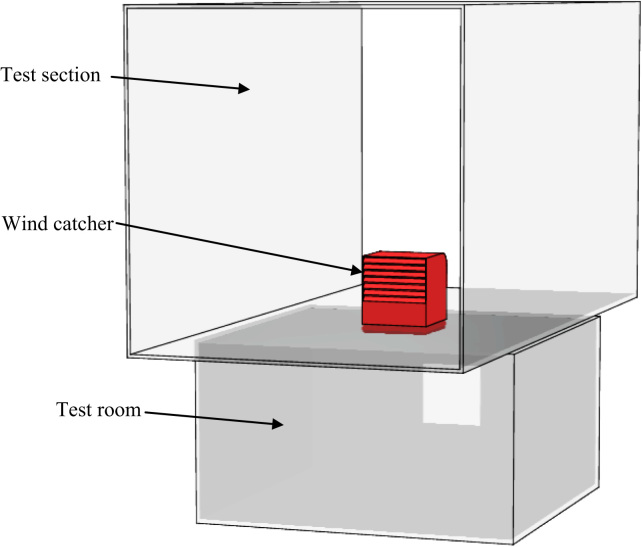
Schematic representation of wind tunnel experiment setup for the wind catcher testing.

Due to the size of the test section, a reduced scale model (1:10) of the wind catcher was used in the experiment. Fig. 1 shows the experimental design for the testing of the wind catcher. The test section represents the outdoor airflow and the indoor was represented by a small room model located at the bottom of the test section. The size of the room was 0.5 m×0.5 m×0.3 m. The room was used to investigate the indoor airflow distribution and also the airflow supplied by the wind catcher device. All the relevant dimensions of the prototype were equally scaled down by the appropriate factor [8], [9].

### 2.2 Materials

#### 2.2.1 Heat pipes

The wind tunnel set-up for heat pipe testing comprised of 19 gravity-assisted wickless heat pipes arranged at 90° vertical to the ground in a staggered grid with a streamwise distance of 20 mm and a spanwise thickness of 50 mm. The heat pipes were manufactured by S&P Coils Ltd. The outer diameter of the copper–water heat pipes was 16 mm with a total length of 800 mm. Water was used as the heat pipe internal working fluid [10], [11]. The heat pipes were charged with water comprising of 2/3rd of the evaporator length, thus indicating a fluid volume of 0.000054 m^3^ [12]. The separator plate was located at the centre of the pipes providing an evaporator and condenser sectional lengths of 400 mm [12].

#### 2.2.2 Wind catcher models

Due to the size of the required wind tunnel model and complexity of several components of the wind catcher geometry, the prototype was created using rapid protoyping. The wind catcher model was connected to a 0.5 m×0.5 m×0.3 m test room, which was mounted underneath the wind tunnel test section. A 0.1 m×0.1 m opening at the leeward wall of the test room represents the outlet. The walls of the test room was made of clear perspex material to allow the accurate positioning of the hot-wire anemometer sensors inside the space. The top plate of the test room was created that it could be rotated in the test section in order to simulate various wind angles.

### 2.3 Measurement method

#### 2.3.1 Heat pipe analysis

The PICO Type K Thermocouple (exposed wire, polytetrafluoroethylene (PTFE) insulated) with a tip diameter of 1.5 mm and a tip temperature range between −75 °C and 250 °C was used for the present experiment. The maximum allowable measurement uncertainty of the device at 0 °C was 0.5 °C while the maximum allowable uncertainty was 0.6 °C at a temperature of 50 °C. The wind tunnel axial fan speed was kept constant at 2.3 m/s to replicate a low Reynolds Number airstream (in the order of 10^3^) typically found in natural ventilation systems. Thermocouple measurement points were located around the physical domain in order to determine the transient formation of heat pipe temperatures when working under convection and conduction principles. Points T1 and T2 were located 0.15 m upstream and downstream of the heat pipes in order to analyse the temperature variation recorded as the external air transfers its heat to the pipes. Simultaneously, points T3 and T4 were located on the central heat pipe, with T3 situated in the evaporator section and T4 situated in the condenser section. Details about the experimental setup are available in [10].

#### 2.3.2 Wind catcher test

The induced airflow into the test room was measured using the hot-wire anemometer, which was located below the channels of the wind catcher. The cross-sectional area of the wind catcher channel was divided into several portions (9 points). Furthermore, the airflow distribution inside the test room was also measured using the hot-wire anemometer. Nine measurement points in an equally spaced 3×3 grid were created within the test room. Additionally, three measurement points were positioned at the bottom of the room (central) and below the supply of the wind catcher. Full details of the location of the points and measurement procedure are available in [1]. The values of the velocity were obtained from the three components of the vector (*X*, *Y*, and *Z*). The experimental tests were carried out at wind velocities between 3 and 5 m/s [13], [14]. These velocities were confirmed during the setup and configuration of the wind tunnel during commissioning. The average was calculated for the period of two minutes, while the results and start or finish times were recorded. The uncertainties associated with the air velocity readings, the hot wire probe (Testo 425) gave velocity measurements with uncertainty of ±1.0% rdg. at speeds lower than 8 m/s and uncertainty of ±0.5% rdg. at higher speeds (8–20 m/s).

## Footnotes

Appendix A Supplementary data associated with this article can be found in the online version at doi:10.1016/j.dib.2015.09.027.
